# Supplementary material for: Identification and evolution of novel cfr-carrying plasmids in XDR Klebsiella pneumoniae strains from a chicken farm
Source: Microbiol Spectr. 2025 Mar 25;13(5):e02628-24. doi: 10.1128/spectrum.02628-24 (PMC12054152; doi:10.1128/spectrum.02628-24)
Supplement: Supplemental material — Fig. S1 to S5. [file spectrum.02628-24-s0001.docx]

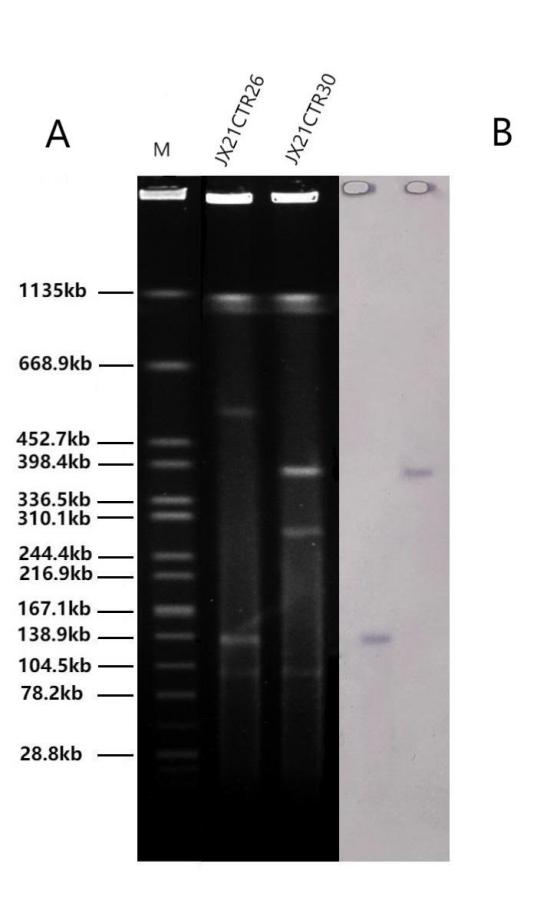


**Figure S1：S1-PFGE and** **Southern hybridization.** S1-PFGE (A) and Southern hybridization (B) of *cfr*-positive *K. pneumoniae* strains J21CTR26 and J21CTR30, with the *cfr* as probe. M, *Salmonella Braenderup* H9812.

**
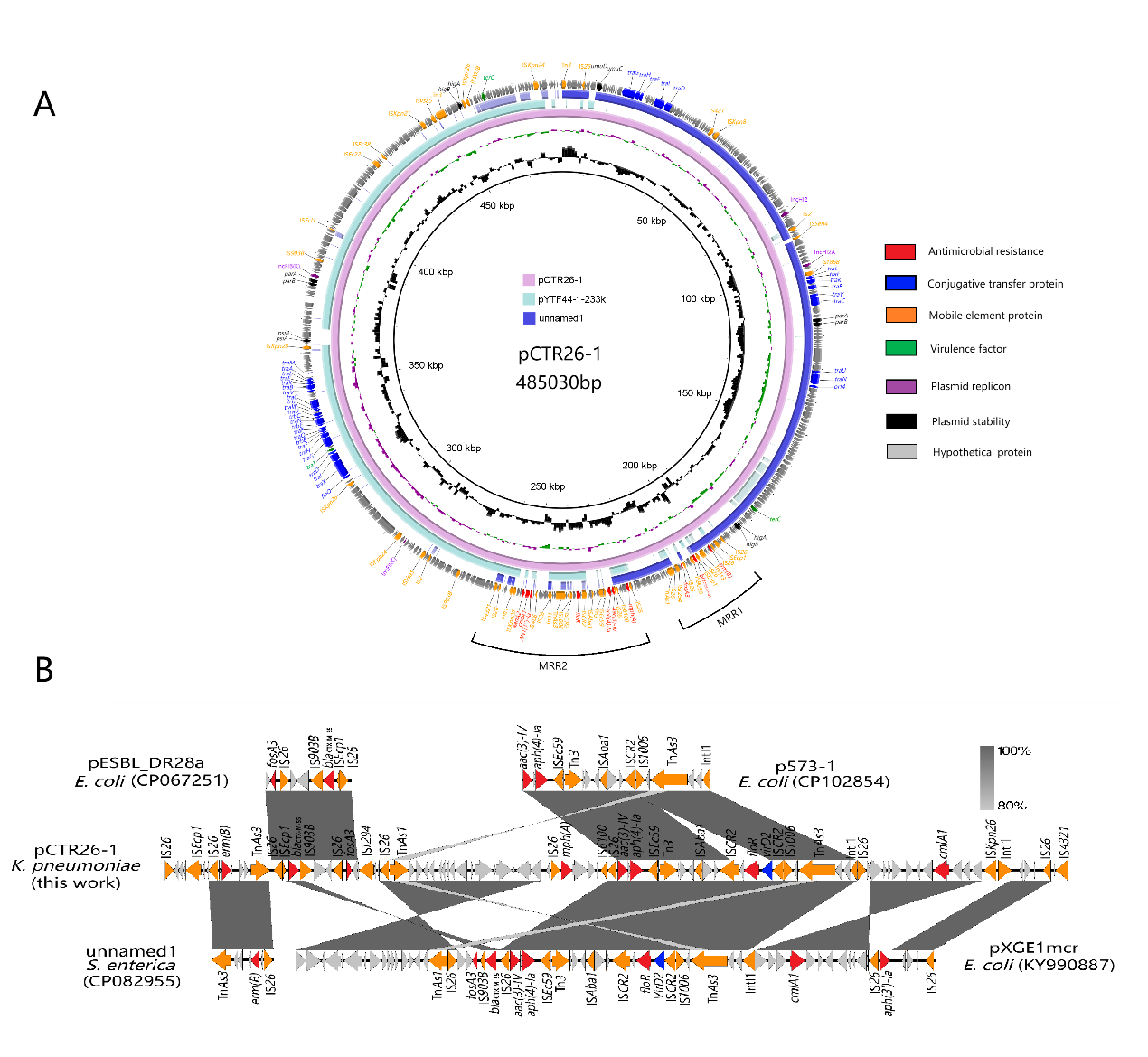
**

**Figure S2: Comparative analysis of pCTR26-1 (A) and the multidrug resistance regions (B).** Blue, red, purple, orange, green, black, and gray arrows represent transfer-related proteins, resistance genes, replicons, mobile elements, virulence genes, plasmid stability proteins, hypothetical and unclassified elements, respectively, and transcription direction is indicated.

**
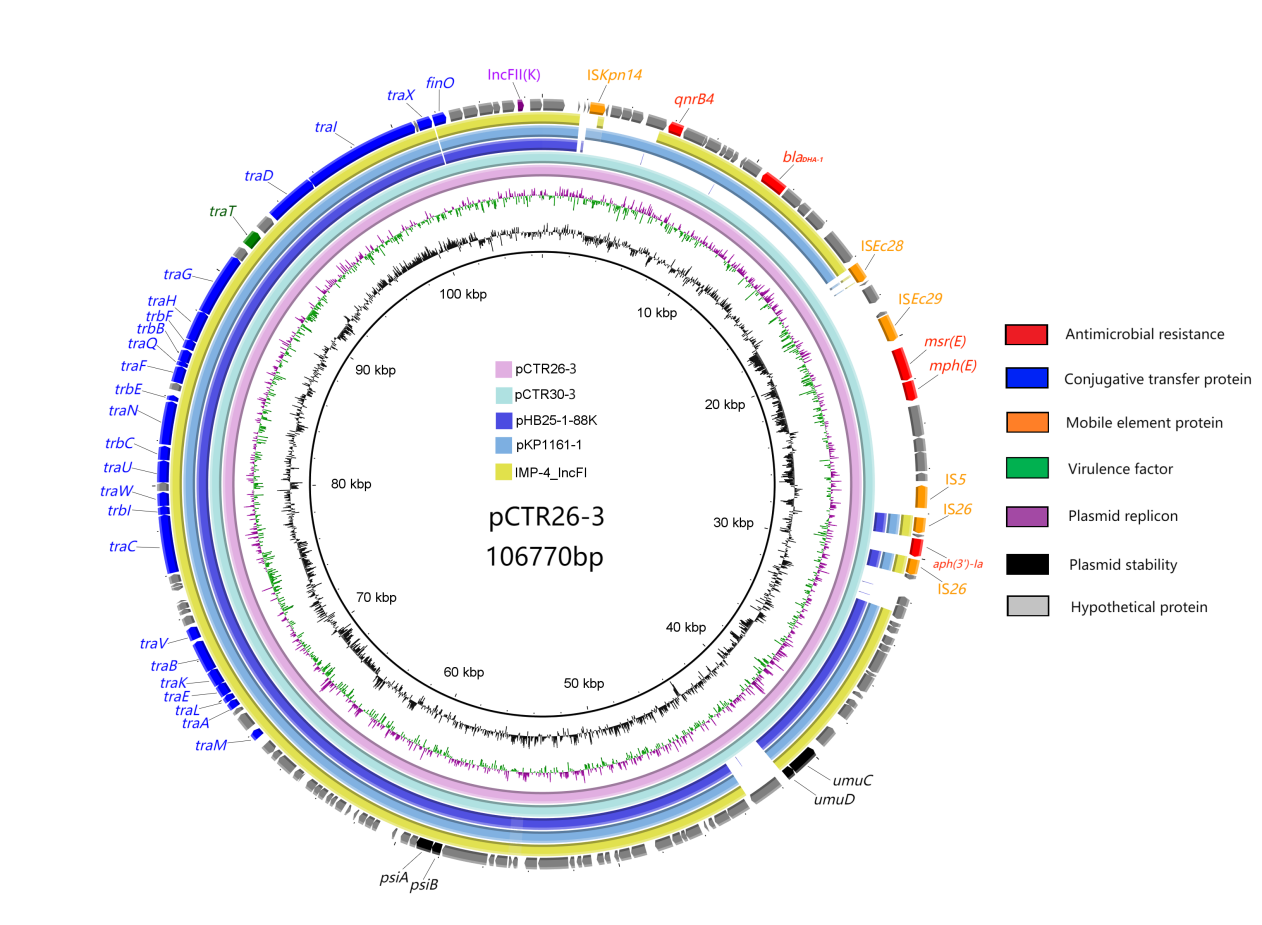
**

**Figure S3: Structural comparisons of plasmids pCTR26-3 and pCTR30-3 in this study and the similar plasmids in GenBank**. Blue, red, purple, orange, green, black, and gray arrows represent transfer-related proteins, resistance genes, replicons, mobile elements, virulence genes, plasmid stability proteins, hypothetical and unclassified elements, respectively, and transcription direction is indicated.


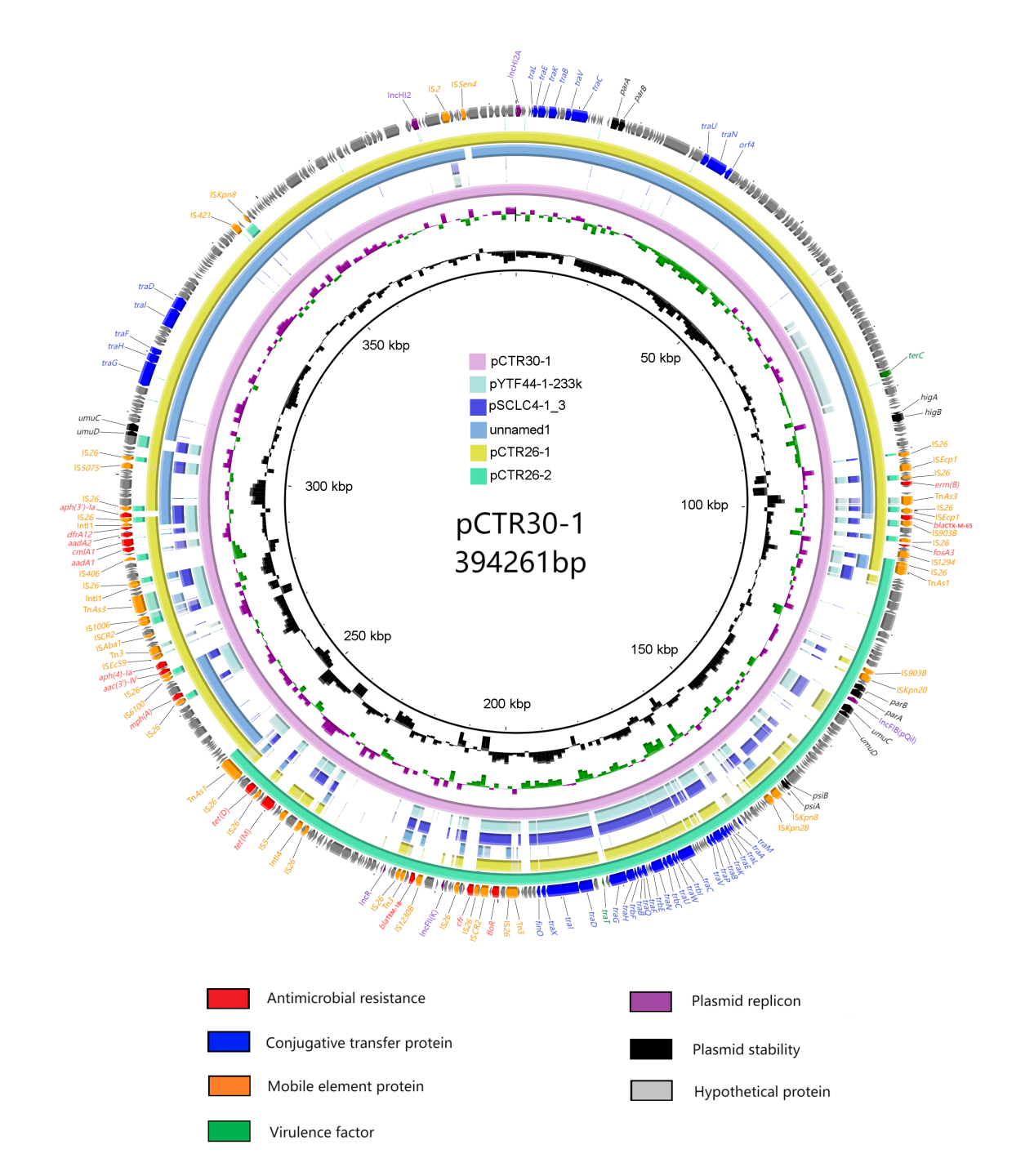


**Figure S4: Structural comparisons of the *cfr*-bearing plasmid** **pCTR30-1 with the similar plasmids.** Blue, red, purple, orange, green, black, and gray arrows represent transfer-related proteins, resistance genes, replicons, mobile elements, virulence genes, plasmid stability proteins, hypothetical and unclassified elements, respectively, and transcription direction is indicated.

**
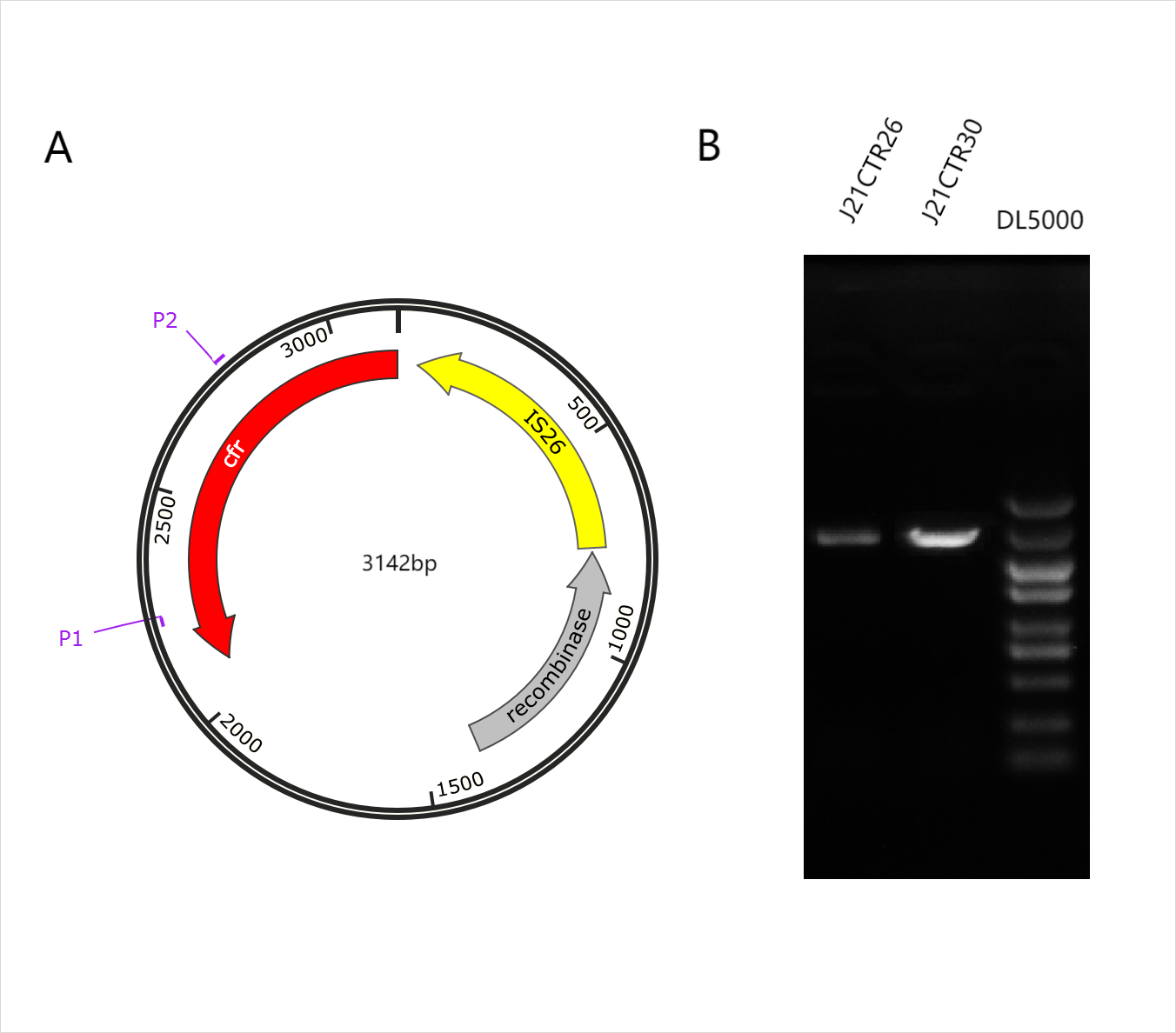
**

**Figure S5：**Structure of circular intermediate IS*26*-*cfr* in J21CTR26 and J21CTR30. (A) Structural diagram of the circular intermediate formed by the *cfr* gene. (B) Size of *cfr*-carrying circular intermediates amplicons obtained by gel electrophoresis. DL5000 stands for 5000bp DNA Marker. P1 and P2 are primer binding sites.
